# Role of ultrasound and magnetic resonance imaging in the prognosis and classification of muscle injuries in professional football players: correlation between imaging and return to sport time

**DOI:** 10.1007/s11547-021-01396-y

**Published:** 2021-07-26

**Authors:** Christian Ossola, Marco Curti, Marco Calvi, Sofia Tack, Stefano Mazzoni, Lucio Genesio, Massimo Venturini, Eugenio Annibale Genovese

**Affiliations:** 1 Department of Diagnostic and Interventional Radiology, University of Insubria, Varese, Italy; 2 Istituto Radiologico “Deriu”, Cagliari, Italy; 3 Medical Doctor Milan AC, Milano, Italy; 4 Medical Clinical Institute Intermedica - Columbus, Milan, Italy; 5 University of Insubria Varese, Varese, Italy

**Keywords:** Soccer, Prognosis, Return to sport, Muscular diseases, Lower extremity

## Abstract

**Purpose:**

To study distractive muscle injuries applying US and MRI specific classifications and to find if any correlation exists between the results and the return to sport (RTS) time. The second purpose is to evaluate which classification has the best prognostic value and if the lesions extension correlates with the RTS time.

**Methods:**

A total of 26 male, professional soccer players (age 21.3 ± 5.6), diagnosed with traumatic muscle injury of the lower limbs, received ultrasound and MRI evaluation within 2 days from the trauma. Concordance between US and MRI findings was investigated. The relationships between MRI and US based injury grading scales and RTS time were evaluated. Correlation between injuries’ longitudinal extension and RTS time was also investigated.

**Results:**

The correlation between US and MRI measurements returned a Spearman value of *r*_*s*_ = 0.61 (*p* = .001). Peetrons and Mueller-Wohlfahrt grading scales correlations with RTS time were *r* = 0.43 (*p* = .02) and *r* = 0.83 (*p* =  < .001). The lesion’s extension correlation with RTS time was *r* = 0.63 (*p* < .001). The correlation between the site of the lesion and its location with the RTS time were *r*_*s*_ = 0.2 and *r*_*s*_ = 0.25.

**Conclusions:**

Both US and MRI can be used as prognostic indicators along with the Peetrons (US) and the Mueller-Wohlfahrt (MRI) classifications. MRI is more precise and generates more reproducible results. The lesion craniocaudal extension must be considered as a prognostic indicator, while the injury location inside the muscle or along its major axis has doubtful significance.

## Introduction

In sports which require high physical effort, in which muscles are highly strained for short periods, such as football, muscle injuries are very common. In a team of professional players, it has been estimated that more than 30% of all injuries are represented by muscle injuries [1–3].

With proper diagnosis, treatment and rehabilitation, the recovery time is shortened and there is a lower risk of reinjury. This objective assumes considerable importance in high-level teams, in which the absence of a player from competition implies strategic burden and has considerable economic implications [2–6].

Several classifications of muscle injury have been proposed, based both on imaging and clinical findings. The focus of each one is different and can involve the severity of the injury, the location and/or the etiology [1, 3, 7].

Magnetic resonance imaging (MRI) and ultrasonography (US) play an important role not only in the identification of the lesion, but also in the determination of its location, extension, severity and, therefore, prognosis [1, 6–8].

At the present time, there are several studies in the literature which try to identify if any radiological parameter can correlate with the recovery time [9–14].

Some factors showed correlation with the risk of reinjury, such as the age of the athlete and a previous injury, considering in particular the hamstring muscle group [15].

Among the MRI parameters, the length and volume of the lesion, the affected area in the horizontal section, and the grading of the lesion were proposed. Even in this case, the hamstring is the most studied muscle group [2, 3, 5, 15–18].

The objectives of this study are to classify distractive muscle injuries applying US and MRI specific classifications and to find if any correlation exists between the results and the return to sport (RTS) time. We also aim at evaluating which classification has the best prognostic value and if the lesions extension or location correlate with the RTS time.

## Methods

Our study was conducted prospectively. Informed consent was obtained from each patient included in the study. The study protocol conforms to the guidelines of the Declaration of Helsinki of 1975.

### Participants

Professional athletes with acute lower limb pain caused by indirect trauma were included in the study between February 2018 and December 2019. For initial eligibility, athletes were required to meet the following inclusion criteria: male sex, clinical diagnosis of acute muscle injury and with the possibility of completing the follow-up until the actual return to the field.

Exclusion criteria were lesions requiring urgent surgery, grade 4 (Mueller-Wohlfahrt) muscle tear (with complete avulsion injury), concomitant fracture, double lesion, and MRI contraindications.

### MRI parameters

All images were obtained using a 1.5-Tesla magnet system (Philips Ingenia Ambition/Elition) with a body matrix coil.

Each patient was evaluated with Coronal STIR TSE (TR 2700-6000, TE 90, TI 140 ms, FOV 400-450 × 400, Slice thickness 4 mm, Matrix 328 × 310, ETL 6, TURBO FACTOR 20, NSA 2, SENSE reduction factor 2); Axial TSE dual proton density-weighted SPAIR and without fat suppression (TR 3000-4000, TE1 5.7 ms, TE2 80 ms, FOV 400X300, Slice thickness 4 mm, Matrix 400 × 250, TURBO FACTOR 18, NSA 2, SENSE reduction factor 2); Axial T1 TSE (TR 520, TE 18, FOV 400X300, Slice thickness 4 mm, Matrice 400 × 250, TURBO FACTOR 5, NSA 2, SENSE reduction factor 2); Axial DWI (b = 0 - 450 - 900)(TR 1759, TE 80-90, FOV 450X400, Slice thickness 4 mm, Matrix 152 × 133, NSA 4, SENSE reduction factor 2).

### MRI and US assessment

One musculoskeletal radiologist (EAG), with more than 15 years of experience in musculoskeletal MRI analyzed and independently reviewed all the MRIs from the athletes initially included twice, blinded to clinical status.

The images were evaluated at two weeks’ distance to reduce recognition bias. A six-hour period between ultrasound evaluations was chosen for logistical reasons of the sports federations. Repeating the ultrasound study at an excessive distance could have affected the repeatability of the study due to the progression of the reparative processes, particularly in minor injuries.

One musculoskeletal radiologist (MC) carried out US examinations twice from 2 to 24 h before or after the MRI examinations. The studies were performed at six hours’ distance minimum to reduce recognition bias.

Each US examination was carried out with a standardized procedure as described by Takebayashi et al. [19]with a MyLab ClassC Advanced system (Esaote Biomedica, Genoa, Italy) using linear transducers with frequencies between 7.5 and 13 MHz. Each MRI and US was reviewed with a standardized scoring form.

Quantitative assessment of the maximal extent of the lesions was performed, including measurement (mm) of the craniocaudal extent of the increased signal intensity on DWI volume reconstructed sequences [11]. All lesions were measured in DWI sequences with a b value of 900.

Only the longitudinal extent of the lesion was considered as it has been proven to be one of the parameters that best correlates with prognosis [11].

Injuries located at the interface between muscle fibers and the proximal and distal aponeuroses/tendons of the muscle bellies were classified as myotendinous injuries, while injuries located at the interface between muscle and fascia were classified as myofascial injuries.

The modified Peetrons comprises four injury severity categories: grade 0 indicates negative MRI without any pathology; grade 1 edema without architectural distortion; grade 2 architectural distortion indicating a partial tear; grade 3 total muscle or tendon rupture. [7, 12, 20]

The original version of Mueller-Wohlfahrt classification was used (Table 1) [3]. Lesions extension was measured in DWI volume reconstructed images in craniocaudal direction. Since the grading categories may overlap due to the different measurements of high signal changes, if any characteristics of a higher-grade injury were present, the injury was scored with the highest grade.

**Table 1:** Mueller-Wohlfahrt classification summary [3]

A. Indirect muscle disorder/injury Muscle disorder	Functional muscle disorder	Type 1: Overexertion-related muscle disorder	Type 1A: Fatigue-induced muscle disorder Type 1B: Delayed-onset muscle soreness (DOMS)
		Type 2: Neuromuscular muscle disorder	Type 2A: Spine-related neuromuscular Muscle disorder Type 2B: Muscle-related neuromuscular
	Structural muscle injury	Type 3: Partial muscle tear	Type 3A: Minor partial muscle tear Type 3B: Moderate partial muscle tear
		Type 4: (Sub)total tear	Subtotal or complete muscle tear Tendinous avulsion
B. Direct muscle injury		Contusion / Laceration	

### Intra-rater reliability

The intra-reader reliability for (EAG) and (MC) was measured using the Cohen’s Kappa coefficient.

### Treatment and time to RTS

Athletes included in the prospective case series received either a similar rehabilitation program, or individualized rehabilitation at the club or federation.

Time to RTS was defined as the number of days from injury until the athlete was cleared to resume unrestricted training by the treating physician or physiotherapist at the club or federation.

The RTS decision was not blinded to the MRI findings.

The number of days until RTS was provided by the club medical staff through weekly phone calls or emails.

### Statistical analyses

Continuous data were tested for normality and presented as average (± standard deviation (SD); range). Categorical data were presented as frequency (%).

Intra-reader agreement between the MRI and US assessments were analyzed through cross-tabulation computing Cohen’s kappa statistic (*ĸ*); standard error (SE) and confidence interval (CI) were also calculated.

Correlation between the scores obtained by MRI and US findings was tested calculating the Spearman’s Rho correlation coefficient.

Correlation between lesions’ extension and location and RTS time was also tested calculating the Spearman’s Rho correlation coefficient.

To measure the correlation between the modified Peetrons (US), Chan and Mueller-Wohlfahrt (MRI) scoring systems and time to RTS, the Pearson correlation coefficient was calculated, if assumptions were met, and nonparametric analyses (Spearman’s Rho) otherwise. The T-Student test was used to test if RTS times between myofascial and myotendinous were different.

A *p* value < 0.05 was considered statistically significant, and exact p values are reported. The data analysis for this paper was generated using the Real Statistics Resource Pack software (Release 7.2). Copyright (2013–2020) Charles Zaiontz. www.real-statistics.com.”

## Results

### Patient characteristics

**Fig. 1:**
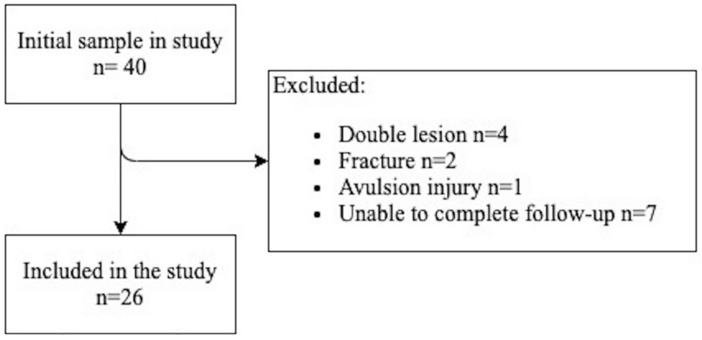
Patients selection flowchart

A total of 26 consecutive patients (age 21.48 ± 5.5; 14–30) matched the eligibility criteria as described in Fig. 1. Patients characteristics are summarized in Table 2. No patients were excluded due to MRI contraindications.

**Table 2:** Patient and injury characteristics

Number of patients *(n)*	26
Age average *(*± *SD; range) mean*	21,48 *(*± *5,5; 14–30) 19*
Days to RTS average *(*± *SD; range), mean*	27.7 *(*± *18; 4–67) 24*
Injured muscle (%)	
Adductor longus	3.85
Semitendinosus	3.85
Semimembranosus	19..23
Rectus femoris	19.23
Biceps femoris	23.08
Ileopsoas	15.38
Soleus	3.85
Medial gastrocnemius	11.54
Anatomical distribution (%)	
Myotendinous junction	76.92
Myofascial junction	19.23
Intramuscular	3.85

### Lesion classification

According to the Peetrons’ classification, 10 lesions were classified as structural and 16 were defined as functional.

A total of 14 lesions were classified as 3A according to Mueller-Wohlfahrt and 9 were judged as 3B. In the remaining cases, lesions were classified as 1b. The hamstrings were more frequently affected (12/26, 46%). Among them, the biceps femoris was the most frequently involved muscle (6/12, 50%), followed by the semimembranosus (5/12, 41.7%) and the semitendinosus (1/12, 8.3%).

The MRI intra-reader agreement calculated with the Cohen’s kappa statistic test resulted in a *ĸ* of 0.86 (SE = 0.09; CI = 0.62–1.10). The US examinations intra-reader agreement resulted in a *ĸ* of 0.79 (SE = 0.1; CI = 0.58–1.0). The correlation between the US and MRI measurements returned a Spearman value of *r*_*s*_ = 0.61 (*p* = .001).

The correlation between the Peetrons and Mueller-Wohlfahrt’s grading scales and the RTS time returned Pearson values, respectively, of *r* = 0.43 (*p* = 0.02) and *r* = 0.83 (*p* =  < 0.001).

The correlation between the RTS time and the initial extension of the lesion measured on MR images in coronal scans was *r* = 0.68 (*p* < 0.01) (Table 3).

**Table 3:** Summary table with correlation of lesion sites, classification, RTS, and lesion length

Patient	Age	Injury location	Limb	Peetrons	Mueller-Wohlfahrt	Site	Return to play time (days)	Lesion CC length
1	16	rectus femoris	L	grade I	type 3A	Muscular-myofascial middle third	20	18
2	16	Iliopsoas	L	grade II	type 3B	Intramuscular-middle third	34	12
3	18	rectus femoris	R	grade II	type 3B	Proximal myotendinous junction	32	21
4	27	semimembranosus	L	grade II	type 3B	Proximal myotendinous junction	62	155
5	17	Iliopsoas	L	grade II	type 3B	Distal myotendinous junction	67	100
6	19	biceps femoris	L	grade I	type 3A	Muscular-myofascial middle third	10	12
7	30	medial gastrocnemius	R	grade I	type 3A	Muscular-myofascial middle third	23	20
8	18	longus adductor	L	grade 0	type 1B	Proximal myotendinous junction	5	7
9	19	Soleus	R	grade I	type 3A	Distal myotendinous junction	12	10
10	17	biceps femoris	L	grade II	type 3B	Proximal myotendinous junction	54	26
11	14	biceps femoris	L	grade 0	type 1B	Proximal myotendinous junction	4	11
12	30	Iliopsoas	R	grade I	type 3A	Distal myotendinous junction	40	83
13	29	rectus femoris	R	grade I	type 3A	Proximal myotendinous junction	20	83
14	19	biceps femoris	L	grade I	type 3A	Proximal myotendinous junction	10	4
15	30	medial gastrocnemius	R	grade II	type 3B	Distal myotendinous junction	45	125
16	26	biceps femoris	R	grade I	type 3A	Muscular-myofascial inferior third	25	35
17	29	rectus femoris	L	grade II	type 3B	proximal myotendinous junction	45	147
18	19	semimembranosus	R	grade I	type 3A	Muscular-myofascial middle third	10	23
19	17	semimembranosus	R	grade II	type 3B	Proximal myotendinous junction	41	46
20	17	Iliopsoas	L	grade I	type 3A	Distal myotendinous junction	34	160
21	16	biceps femoris	R	grade I	type 3A	Proximal myotendinous junction	8	15
22	25	semimembranosus	L	grade I	type 3A	Proximal myotendinous junction	23	70
23	30	medial gastrocnemius	R	grade II	type 3B	Distal myotendinous junction	45	180
24	19	rectus femoris	R	grade 0	type 1B	Proximal myotendinous junction	5	10
25	19	semimembranosus	L	grade I	type 3A	Proximal myotendinous junction	26	20
26	17	semitendinosus	R	grade I	type 3A	Distal myotendinous junction	21	11

The correlation between the site of the lesion and its location with the RTS time returned Spearman values, respectively, of *r*_*s*_ = 0.2 and *r*_*s*_ = 0.25. The comparison between the mean RTS times in myofascial and myotendinous injuries resulted in t = − 1.37055 (*p* = 0.091871).

## Discussion

Predicting the correct RTS time is fundamental for professional players. This is namely true for professional football players, for whom a muscular injury implies strategic and financial issues [2, 3]. Moreover, a premature RTS can result in reinjury [21]. The UEFA Champions League (UCL) Injury Study, for example, analyzed the frequency of injuries occurred in 12 years in the UCL teams [17, 21].

Among the proposed parameters to predict the RTS time, most of the practitioners refer to the MRI parameters, such as length and volume of the lesion, location of the affected area and grading of the lesion, especially for lesions involving the hamstrings [2, 5, 15–18, 22].

In our sample, the lesion distribution was mostly coherent with that described in the literature: the hamstrings were more frequently affected by distractive lesions (12 lesions of 26, 46%), with the biceps femoris being involved the most frequently (6 of 12, 50%). The only difference was the higher prevalence of semimembranosus injuries (5 of 12, 41.7%) followed by the semitendinosus (1 of 12, 8.3%) [22].

Comparing the mean recovery time in our experience with those proposed by Maffulli and others (17), we found coherent results. Injuries to the adductor muscles, in fact, required a shorter recovery time, followed by the group of flexors, femoral quadriceps and calf muscles [5, 17]. A negative MRI correlated with a shorter recovery time (6–9 days).

Considering the Mueller-Wohlfahrt classification, the proposed stop intervals in the literature should be 5 to 15 days for functional lesions (i.e., 1a, 1b, 2a, 2b), 15 to 18 days for 3a and 25 to 35 days for 3b structural lesions.

In our study, the “stop time” ranged between 4 and 67 days. 5/26 cases (19%) were perfectly overlapping in regard to the clinical-radiological healing time, 10/26 (39%) differed by ± 5 days compared to the expected outcome, and the remaining 11 cases (42%) diverged in a time frame ranging between + 7 and + 32 days.

The reason for this discrepancy may be due to the small sample size or due to biases introduced for tactical reasons.

Diagnostic over-grading associated with cautious rehabilitation may be other reasons.

The lesion craniocaudal extension correlates with the RTS duration. In particular, both the Peetrons’ (US) and the Mueller-Wohlfahrt’s (MRI) classifications demonstrated positive correlation, the latter with stronger significance than the former [3, 7].

As a result, both the classifications should have a prognostic value, even if it must be taken into account that ultrasonography tends to be less reproducible. We must analyze this result after obtaining a “good” to “excellent” intra-reader reliability in US examinations and an “excellent” to “almost perfect” intra-reader reliability in MRI examinations.

In the literature, the attitudes about the validity of ultrasonography as a prognostic factor are conflicting.

**Fig. 2:**
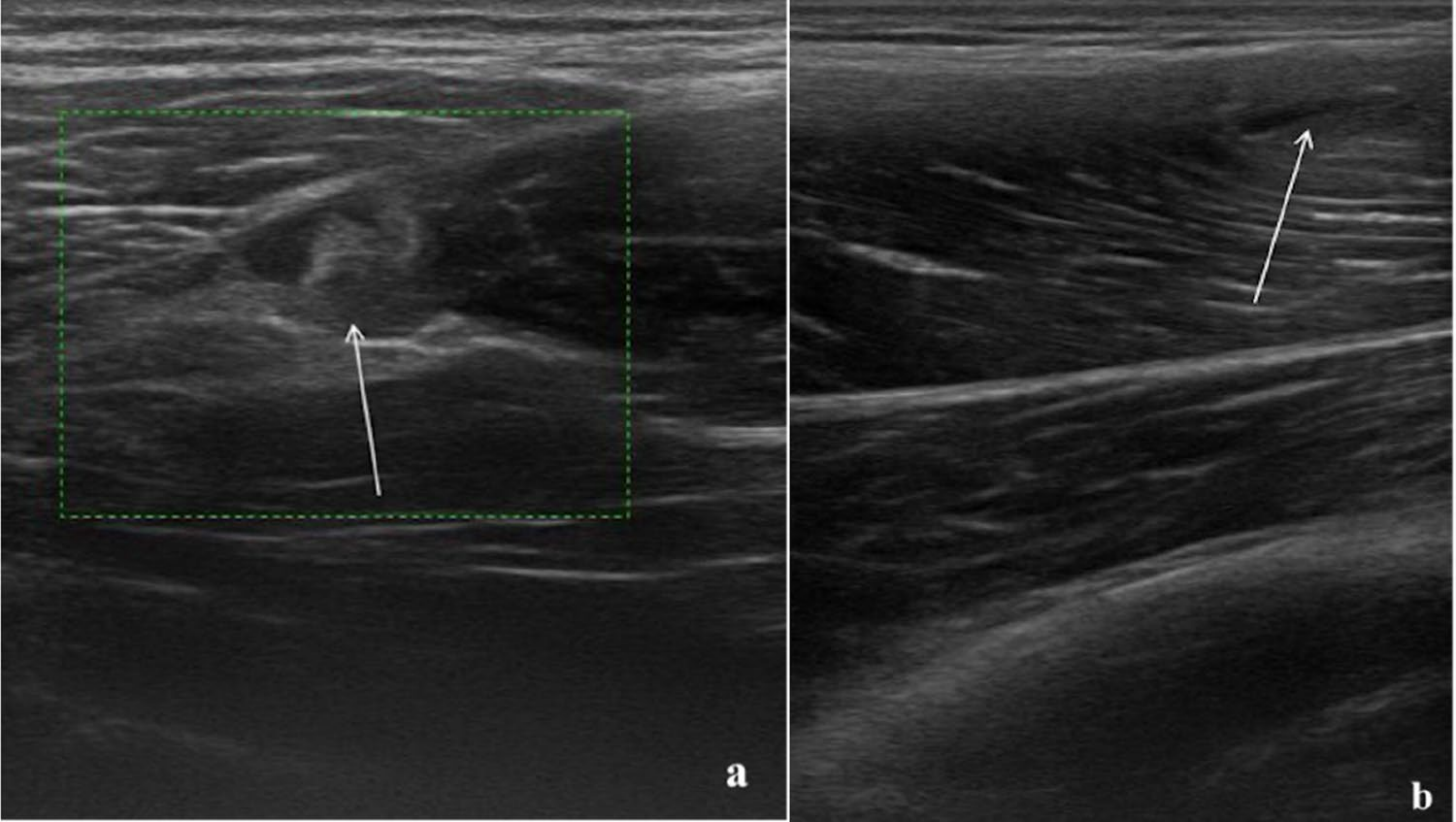
**a** 24-year-old athlete with type 2 muscle tear (Peetrons). The alteration of the echostructure involves the proximal myotendinous junction of the femoral biceps muscle and is characterized by a small interruption in the continuity of the fibers (arrow); **b** 22-year-old athlete with type 2 muscle tear (Peetrons). The US examination illustrates a small myofascial lesion (arrow) in the rectus femoris

Connell and others showed a correlation between the length of the lesion on ultrasonography and recovery time, while Petersen and others argued that there was no correlation with the extent of the lesion and that there were no differences in prognosis between players that showed ultrasonography abnormalities and those who had a normal ultrasound examination (Fig. 2) [15, 23].

**Fig. 3:**
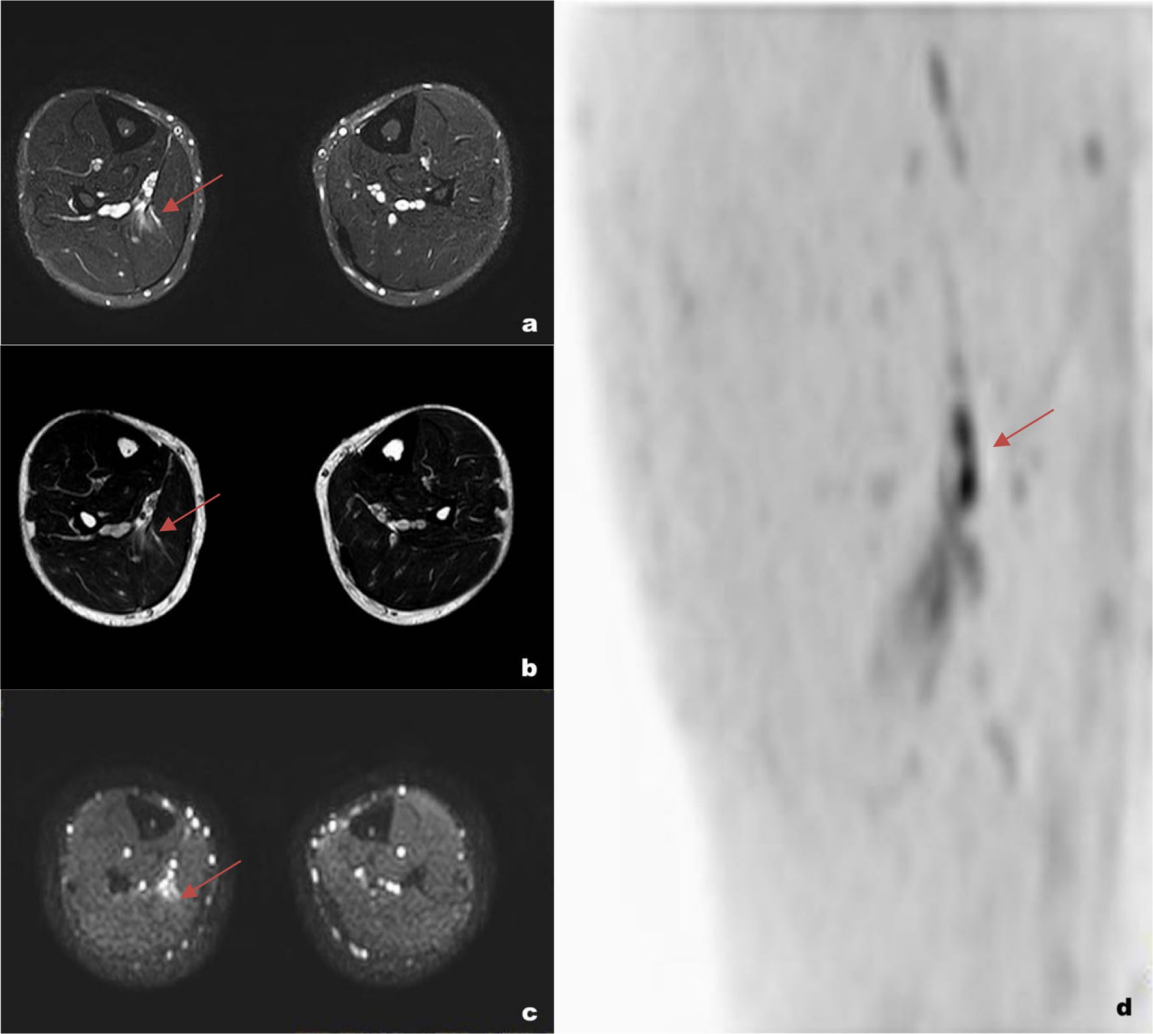
a 26-year-old athlete with a myotendinous lesion in the middle third of the right soleus (red arrow). The Axial TSE dual proton density-weighted SPAIR (**a**) and axial TSE dual proton density-weighted images without fat suppression (**b**) show both intramuscular edema and muscle fibers interruption at the myotendinous junction. The DWI images (**c**) allow a clearer representation of the actual muscular injury (hyperintense spot). The DWI volume reconstruction (d) allows the precise evaluation of lesion craniocaudal extension. The lesion was classified as 3a using the Mueller-Wohlfahrt classification. The value on the ADC map was 1.6·10^–3^ mm^2^/s

In the literature, MRI is considered the technique of choice to guide clinical choices as it can be used to precisely identify and measure the intramuscular edema and the presence of fibro-cicatricial tissue (Fig. 3) [5].

Several studies demonstrated the persistence of hyperintense signal in fluid-sensitive sequences in 36% to 89% of clinically cured athletes. This may imply that the healing process occurs over a rather long period of time, which lasts for weeks or months after the return to the field [5, 24], but may also be the evidence that not every hyperintense area has the same significance and deeper research is required to find more precise prognostic indicators.

The location of the injury (i.e., myofascial, myotendinous, etc.) did not correlate with RTS duration. O’Chan and others highlighted the fact that a lesion involving only a tendon shows a longer recovery time than those involving muscles or myotendinous junctions. Also, Corazza et al. and Connell et al. argued that there are differences in prognosis between myofascial injuries and those involving the myotendinous junction [6, 15].

In our study, this association was not evident and the reason may be the inhomogeneity of the sample, as the majority of the lesions were myotendinous [1].

Similarly, Askling et al. claimed that the location of the lesion in the muscle (i.e., proximal, distal) considering the hamstrings, affects the prognosis and stated that the farer the injury is from the ischial tuberosity the better is the prognosis [1, 18]. In our study, this parameter was not taken into account. A further project could evaluate the results of this study by constructing homogeneous groups of athletes divided according to the distance of the injury from the bone heads to see if there really is a significant difference in prognosis.

The limitations of our study include the fairly small number of patients examined and the fact that our study population was an elite group of professional players. Therefore, our findings may not be strictly applicable to the general population, although the homogeneity of the sample (male players aged 16 and 30) can be considered to be a strong point of our study.

A further limitation regarding the prognosis of individual athletes is the lack of a standardized treatment protocol.

In conclusion, both US and MRI can be used as prognostic indicators along with the Peetrons (US) and the Mueller-Wohlfahrt (MRI) classifications. MRI is more precise and generates more reproducible results.

The lesion craniocaudal extension must be considered as a prognostic indicator, while the importance of the injury location inside the muscle or along its major axis has doubtful significance.
